# Survival of patients with chronic heart failure in the community: a systematic review and meta‐analysis

**DOI:** 10.1002/ejhf.1594

**Published:** 2019-09-16

**Authors:** Nicholas R. Jones, Andrea K. Roalfe, Ibiye Adoki, F.D. Richard Hobbs, Clare J. Taylor

**Affiliations:** ^1^ Nuffield Department of Primary Care Health Sciences University of Oxford Oxford UK; ^2^ Foundation Training Programme Oxford University Hospitals NHS Foundation Trust, John Radcliffe Hospital Oxford UK

**Keywords:** Heart failure, Prognosis, Survival analysis, Systematic review, Meta‐analysis

## Abstract

**Aim:**

To provide reliable survival estimates for people with chronic heart failure and explain variation in survival by key factors including age at diagnosis, left ventricular ejection fraction, decade of diagnosis, and study setting.

**Methods and results:**

We searched in relevant databases from inception to August 2018 for non‐interventional studies reporting survival rates for patients with chronic or stable heart failure in any ambulatory setting. Across the 60 included studies, there was survival data for 1.5 million people with heart failure. In our random effects meta‐analyses the pooled survival rates at 1 month, 1, 2, 5 and 10 years were 95.7% (95% confidence interval 94.3–96.9), 86.5% (85.4–87.6), 72.6% (67.0–76.6), 56.7% (54.0–59.4) and 34.9% (24.0–46.8), respectively. The 5‐year survival rates improved between 1970–1979 and 2000–2009 across healthcare settings, from 29.1% (25.5–32.7) to 59.7% (54.7–64.6). Increasing age at diagnosis was significantly associated with a reduced survival time. Mortality was lowest in studies conducted in secondary care, where there were higher reported prescribing rates of key heart failure medications. There was significant heterogeneity among the included studies in terms of heart failure diagnostic criteria, participant co‐morbidities, and treatment rates.

**Conclusion:**

These results can inform health policy and individual patient advanced care planning. Mortality associated with chronic heart failure remains high despite steady improvements in survival. There remains significant scope to improve prognosis through greater implementation of evidence‐based treatments. Further research exploring the barriers and facilitators to treatment is recommended.

## Introduction

One to two in every 100 adults in the general population, and more than one in 10 people aged over 70 years are diagnosed with heart failure (HF).^1^, ^2^ The true prevalence is likely closer to 4%, as HF often goes unrecognised or misdiagnosed, particularly in older people.^3^, ^4^ Prevalence has risen by almost 25% since 2002 due to factors such as population ageing, improved survival following coronary events and an increase in the prevalence of HF risk factors, including hypertension and atrial fibrillation.^5^ HF is associated with significant morbidity and mortality equivalent to common forms of cancer.^6^

Much existing research on HF prognosis has focused on survival rates for people with 'acute' HF who have been admitted to hospital with a sudden deterioration in symptoms.^7^ An acute decompensation is itself a poor prognostic sign and therefore these survival estimates are not directly applicable to people with 'chronic' HF, who have had an extended period of symptom stability.^7^ Previous research suggests 1‐year survival in acute HF is between 55% and 65%,^8^, ^9^ compared to 80% to 90% in chronic HF.^10^, ^11^

The majority of patients have chronic HF and are treated in ambulatory settings.^12^ This chronic phase should be a time to discuss advanced care planning and anticipated disease progression with patients and their families. These conversations rely on healthcare professionals providing accurate prognostic information, yet survival estimates for chronic HF vary significantly across studies. The pattern of disease progression in HF is also unpredictable and varies considerably between individuals.^13^ Uncertainty over disease trajectory is one reason active HF treatment often persists into the terminal phases of illness, resulting in a large increase in resource use in the last 6 months of life.^14^ It also explains why some clinicians lack confidence in discussing HF prognosis and so avoid the subject.^15^, ^16^ Not all patients wish to know or discuss their prognosis, but for those who do, the ambiguity around their future can be distressing and many would welcome more information.^17^ Where patients are not informed of their expected prognosis, they tend to significantly overestimate their likely life expectancy.^18^

Reliable prognostic estimates can help to promote advanced care planning, improve shared understanding of treatment goals and facilitate integrated treatment with specialist services, including palliative care.^16^ The aim of this systematic review was to assimilate the existing evidence base to provide accurate survival estimates for people with chronic HF. We also aimed to identify key factors which explain the existing variation in prognostic estimates, including age at time of diagnosis, left ventricular ejection fraction (LVEF), decade of diagnosis, and study setting.

## Methods

The protocol was published on PROSPERO (registration number CRD42017075680) and in *Systematic Reviews*.^19^ Reporting adheres to the 'Meta‐analysis Of Observational Studies in Epidemiology' (MOOSE) guidelines (online supplementary *Methods* *S1*).^20^

### Search strategy

We conducted a systematic search of relevant databases from inception to August 2018, incorporating Medical Subject Heading Indexation (MESH) terms and integrated validated search filters from the Scottish Intercollegiate Guidelines Network^21^ (online supplementary *Table* *S1*). A hand search of the included papers' references and relevant review articles was completed to achieve literature saturation.

### Eligibility criteria

Eligible studies reported survival time for adult patients with a diagnosis of HF in the 'chronic' or 'stable' phase.^7^ Survival times were calculated from diagnosis, or from point of study recruitment if this information was unavailable. Studies with under 1‐year follow‐up were excluded given the lack of information on long‐term prognosis. We included studies reporting outcomes for both acute and chronic HF where it was possible to extract survival rates for chronic HF. If the results were combined, we attempted to contact study authors. As our aim was to report survival time in the context of usual care, we excluded interventional studies, service evaluations and studies where participants had been recruited on the basis of another co‐morbidity. Conference abstracts were excluded as having insufficient detail for quality assessment.

### Data analysis

Two authors (N.R.J., I.A.) independently completed two rounds of screening, the first based on titles and abstracts and the second a full text review. Foreign language papers were translated before assessment. Disagreements were checked with a third reviewer (C.J.T.). Two authors (N.R.J., I.A.) also completed independent duplicate data extraction.

Pooled survival rates were calculated at pre‐specified time points using a random effects model given the anticipated variability in study methods. We used the *metaprop* command in Stata 14, designed for meta‐analysis of binomial data.^22^ We calculated the study‐specific 95% confidence intervals using the score statistic via the *cimethod(score)* function and used the *ftt* command to perform the Freeman–Turkey double arcsine transformation and stabilise variance in our weighted pooled estimates.^22^ Heterogeneity and consistency were assessed using Chi‐squared and I^2^ statistics respectively. Sources of heterogeneity were explored using pre‐specified sensitivity and subgroup analyses.

We conducted subgroup analyses and meta‐regression for study date, setting, age and LVEF. To pool study dates, we categorised each included study or relevant subgroup by the decade of participant recruitment. Mean participant age was used to categorise results as either <  65, 65–74 or ≥ 75 years. Study setting was determined by point of recruitment and majority of management. Where there was evidence of significant input across both primary and secondary care, studies were classified as 'cross‐discipline'. HF was categorised as HF with preserved ejection fraction (HFpEF) if LVEF ≥ 50%, HF with mid‐range ejection fraction (HFmrEF) with LVEF in the range 40–49%, and HF with reduced ejection fraction (HFrEF) if LVEF < 40%. Some earlier studies did not include a mid‐range group and so categorised HFpEF as LVEF ≥ 40%. Studies reporting pooled outcomes for all three groups or not measuring LVEF were grouped as 'mixed' ejection fraction. Data were unavailable to allow all subgroups of interest to be included together as covariates in a meta‐regression analysis, therefore each covariate was considered separately in meta‐regression models of survival rates at 1 and 5 years.

Two authors (N.R.J., I.A.) independently completed a risk of bias assessment for each study using the Quality in Prognosis Studies (QUIPS) tool, recommended by the Cochrane Prognosis Methods Group.^23^ We conducted a sensitivity analysis excluding studies at moderate or high risk of bias. We report a Grading of Recommendations Assessment, Development and Evaluation (GRADE) score to provide an estimate of confidence in the cumulative outcomes (online supplementary *Methods* *S2*).^24^

## Results

### Study characteristics

We included 60 studies after screening, 5423 studies at the title and abstract stage and 97 full texts (online supplementary *Figure* *S1*). A number of studies reported survival rates from the same dataset. Where these provided relevant information for our pre‐specified subgroup analyses, we included both studies in the review but only one in any single meta‐analysis. Two studies met the inclusion criteria but reported survival rates at time points which could not be pooled; these are reported narratively.^16^, ^25^

The majority of included studies were conducted in Europe or North America and recruited participants from primary care (*n* = 23), cardiology outpatient clinics (*n* = 20), or both (*n* = 15). Over half were longitudinal cohort studies (*n* = 34) but many recent studies have analysed big databases of routinely collected patient information.^9^ HF diagnosis was most frequently captured using validated database codes (*n* = 19), though many studies also defined HF using Framingham (*n* = 12), or European Society of Cardiology (*n* = 10) criteria (*Table* 1).^1^, ^10^, ^11^, ^25^, ^26^, ^27^, ^28^, ^29^, ^30^, ^31^, ^32^, ^33^, ^34^, ^35^, ^36^, ^37^, ^38^, ^39^, ^40^, ^41^, ^42^, ^43^, ^44^, ^45^, ^46^, ^47^, ^48^, ^49^, ^50^, ^51^, ^52^, ^53^, ^54^, ^55^, ^56^, ^57^, ^58^, ^59^, ^60^, ^61^, ^62^, ^63^, ^64^, ^65^, ^66^, ^67^, ^68^, ^69^, ^70^, ^71^, ^72^, ^73^, ^74^, ^75^, ^76^, ^77^, ^78^, ^79^, ^80^, ^81^ In eight studies the criteria for defining HF was unspecified or relied on a clinical diagnosis. There were insufficient data to conduct a meaningful analysis comparing outcomes by sex.

**Table 1:** Summary of included studies

First author	Year	Study dates	Country	Study setting	Study design	HF definition	Total participants	HF sample	Participants	QUIPS score
Cleland^26^	1987	Not stated	UK	Cardiology outpatient	Prospective cohort	Diagnosis based on clinical, radiological and echocardiogram findings	152	152	Symptomatically stable, NYHA class II–IV	High
Ho^27^	1993	1948–1988	USA	Primary care	Prospective cohort	Framingham criteria	9405	652	Incident HF cases in Framingham and Framingham offspring studies	Moderate
Senni^28^	1998	1991	USA	Cross‐discipline	Routinely collected data	'Slight modification' of Framingham criteria	216	216	Incident HF cases in Rochester Epidemiology Project	Low
McAlister^29^	1999	1989–1995	Canada	Cardiology outpatient	Prospective cohort	Framingham criteria	566	566	Consecutive, confirmed cases of HF at a specialist HF clinic	Moderate
Niebauer^30^	1999	1980–1993	UK	Cardiology outpatient	Prospective cohort	Not defined	99	99	Patients from HF outpatient clinic with very low LVEF (≤20%)	High
Cicoira^31^	2001	1992–1998	UK	Cardiology outpatient	Prospective cohort	Typical symptoms + radiological or clinical evidence of HF.	188	188	Consecutive patients aged >70 years from HF clinic	High
Mosterd^10^	2001	1990–1993, follow‐up to 1996	Netherlands	Primary care	Prospective cohort	Two‐step process involving typical signs, evidence of cardiovascular disease and exclusion of COPD	5255	181	Incident HF cases in Rotterdam Study	Low
Chen^32^	2002	1996–1997	USA	Cross‐discipline	Prospective cohort	Database code of HF, validated using Framingham criteria	83	83	Incident HF cases in Rochester Epidemiology Project, with LVEF >45% and no valve disease	Low
Levy^33^	2002	1950–1999	USA	Primary care	Prospective cohort	Framingham criteria	10 311	1075	Incident HF cases in Framingham study	Moderate
Muntwyler^34^	2002	1999–2000	Switzerland	Primary care	Prospective cohort	ESC and Framingham criteria	411	411	Incident HF cases (NYHA class II–IV) in 'Improvement of HF' primary care survey	Moderate
Ansari^35^	2003	1996	USA	Cardiology outpatient	Retrospective cohort	ICD‐9	403	403	Incident HF cases at Northern California Kaiser Medical Centre	Moderate
Koseki^36^	2003	2000–2001	Japan	Secondary care (mixed)	Registry	LVEF >50%, LVDD >55 mm documented history of congestive HF	721	702	Chronic HF population within regional registry	High
MacCarthy^37^	2003	1993–1995	UK	Cardiology outpatient	Prospective cohort	Typical symptoms and objective evidence of cardiac dysfunction	522	522	Incident, stable, symptomatic HF cases in UK HEART study	Moderate
Nielsen^38^	2004	1993–1996	Denmark	Cross‐discipline	Prospective cohort	Typical symptoms or an abnormal chest X‐ray and current prescription for a loop diuretic	2157	115	Incident cases of HF from four general practices	Moderate
Bleumink^1^	2004	1989–1993 follow‐up to 2000	Netherlands	Primary care	Prospective cohort	Validated score based on ESC criteria	7734	725	Incident HF cases in Rotterdam Study	Moderate
Raymond^39^	2004	1997–2000	Denmark	Primary care	Prospective cohort	ESC criteria	764	36	Volunteer sample from select GPs screened for HF	Low
Roger^40^	2004	1979–2000	USA	Primary care	Prospective cohort	ICD‐9‐CM, validated with Framingham criteria	4537	4537	Incident HF cases in Rochester Epidemiology Project	Low
Cacciatore^41^	2005	1992–2003	Italy	Primary care	Prospective cohort	Medical note review and physical examination to confirm cases, categorised by NYHA status	1259	120	Random sample of elderly patients enrolled in the Southern Italy community cohort	Moderate
Senni^42^	2005	1995 and 1999	Italy	Cardiology outpatient	Routinely collected data	Framingham criteria	1315	1315	The 'IN‐CHF' National Registry of elderly cardiology outpatients with HF	Low
Barker^43^	2006	1970–1974 and 1990–1994	USA	Cross‐discipline	Routinely collected data	Framingham criteria	40 671	1942	Incident HF cases amongst Kaiser Northwest Region health‐plan members	Moderate
van Jaarsveld^44^	2006	1993–1998	Netherlands	Primary care	Prospective cohort	International classification of primary care criteria	5279	293	Incident HF cases in Groningen Longitudinal Aging Study (GLAS)	Moderate
Tsutsui^45^	2007	2004–2005	Japan	Cross‐discipline	Registry	Framingham criteria	2685	2685	Prospective multicentre JCARE‐GENERAL HF registry, including primary care and outpatient data	Low
Ammar^46^	2007	1997–2000	USA	Cross‐discipline	Prospective cohort	American College of Cardiology, American Heart Association definitions	2029	244	Incident HF cases in Rochester Epidemiology Project	Moderate
Hobbs^47^	2007	1995–1999 follow‐up to 2004	UK	Primary care	Prospective cohort	ESC criteria	6162	449	Randomly sampled from four discrete primary care populations and screened for LVSD and HF	Low
Huang^48^	2007	1991–1993	Taiwan	Primary care	Prospective cohort	Framingham criteria	2660	147	Incident HF cases amongst volunteer community sample	Moderate
Curtis^49^	2008	1994–2003	USA	Cross‐discipline	Routinely collected data	ICD‐9‐CM	622 786	622 786	Incident HF cases amongst Medicare patients	Moderate
Henkel^50^	2008	1979–2002	USA	Cross‐discipline	Prospective cohort	ICD‐9 CM	1063	1063	Incident HF cases in Rochester Epidemiology Project	Low
Castillo^51^	2009	1999–2003	Spain	Cardiology outpatient	Registry	Clinician decided. No stated diagnostic criteria	4720	1416	Patients with confirmed HFpEF within the BADAPIC registry	Low
Goda^52^	2009	2004–2005	Japan	Secondary care (mixed)	Prospective cohort	Diagnosis based on clinical, radiological and echocardiogram findings. No stated diagnostic criteria	4255	597	Incident HF cases, NYHA class II–IV, at The Cardiovascular Institute Hospital, Tokyo	Moderate
Parashar^53^	2009	1989–1993	USA	Primary care	Prospective cohort	Individual clinician diagnosis and on active HF treatment	5888	1264	Incident cases of HF within the Cardiovascular Health Study	Low
Jimenez‐Navarro^54^	2010	2000–2003	Spain	Cardiology outpatient	Registry	ESC criteria	4720	4720	BADAPIC registry across 62 centres with HF specific unit	Moderate
Devroey^55^	2010	2005–2006	Belgium	Primary care	Prospective cohort	Individual clinician diagnosis	754	557	Incident HF cases from 178 sentinel GPs	High
Pons^56^	2010	2001–2008	Spain	Cardiology outpatient	Prospective cohort	Not stated	960	960	Consecutive referrals to specialist HF unit	High
Gomez‐Soto^57^	2011	2000–2007	Spain	Cross‐discipline	Prospective cohort	Framingham criteria	4793	4793	Incident HF cases amongst all residents in region of Southern Spain	Low
Grundtvig^58^	2011	2000–2006	Norway	Cardiology outpatient	Prospective cohort	Typical symptoms + radiological or clinical evidence of HF	3632	3632	Incident cases of HF from 24 outpatient clinics	Low
Yeung^59^	2012	1997–2007	Canada	Cross‐discipline	Routinely collected data	ICD‐9/ICD‐10 code	5 175 179	203 361	Incident cases of HF within the Ontario Health Insurance Plan database	Low
Taylor^60^	2012	1995–1999 follow‐up to 2009	UK	Primary care	Prospective cohort	ESC criteria	6162	449	Random sample from 16 socio‐economically diverse GPs screened for HF	Low
Fragasso^61^	2013	1992–2005	Italy	Cardiology outpatient	Routinely collected data	ESC criteria	372	372	Consecutive HF outpatient clinic patients with LVEF <45%	Moderate
Frigola‐Capell^62^	2013	2005–2007	Spain	Primary care	Retrospective cohort	ICD‐10‐GM	13 008	5659	Combined data from urban and rural primary care units in Catalonia, Spain	Low
Gupta^63^	2013	1993–1995	USA	Primary care	Prospective cohort	Gothenburg criteria or ICD‐9 code	1962	116	Incident HF cases amongst middle‐aged African American people within ARIC study	Low
Maggioni^64^	2013	2009–2010	12 European countries	Cardiology outpatient	Prospective cohort	Clinical diagnosis by individual clinicians	5118	4118	Incident HF cases in EURObservational Programme	Moderate
Zarrinkoub^65^	2013	2006–2010	Sweden	Cross‐discipline	Routinely collected data	ICD‐10 code	88 038	88 038	Incident HF cases within Stockholm Health Registry	Low
Singh^25^	2014	2002–2007	UK	Cardiology outpatient	Retrospective cohort	Modified ESC criteria	1041	513	Consecutive patients referred to HF assessment clinic ‐ the Darlington Retrospective outpatient study (DROPSY)	Moderate
Stalhammar^66^	2014	2005–2006	Sweden	Primary care	Retrospective cohort	ICD‐10 codes	137	137	Incident cases of HF with LVEF >50% in 31 primary care centres	Moderate
James^67^	2015	2002–2012	Ireland	Cardiology outpatient	Routinely collected data	Typical symptoms, raised BNP and echocardiogram changes	733	285	Consecutive primary care referrals to Rapid Access Clinic for suspected HF (NYHA class II–III)	Moderate
Sarria‐Santamera^68^	2015	2006–2010	Spain	Primary care	Retrospective cohort	ICD‐10 codes	227 984	3061	HF codes on primary care database	Low
Crespo‐Leiro^69^	2016	2011–2013	12 European countries	Cardiology outpatient	Registry	ESC criteria	12 440	12 440	Long‐term HF prospective registry across 21 European countries	Moderate
Akwo^70^	2017	2002–2010	USA	Primary care	Prospective cohort	ICD‐9 codes	27 078	4341	Incident HF cases in Southern Community Cohort Study	Low
Al‐Khateeb^71^	2017	2000–2015	Saudi Arabia	Cardiology outpatient	Retrospective cohort	Clinical diagnosis + LVEF <45%	2298	2298	Consecutive patients seen in HF clinic, with LVEF <45%	Moderate
Dokainish^72^	2017	2012–2014	International	Cardiology outpatient	Prospective cohort	Clinical diagnosis by individual clinicians	5823	5823	Consecutive sample of outpatients and inpatients with HF across six regions	High
Farre^73^	2017	2012	Spain	Cross‐discipline	Registry	ICD‐9‐CM	88 195	88 195	Longitudinal study of all prevalent cases of HF within Catalonian public health database	Low
Farre^74^	2017	2001–2015	Spain	Cardiology outpatient	Prospective cohort	ESC criteria	3580	3580	Consecutive sample from four HF units	Low
Koudstaal^75^	2017	1997–2010	UK	Primary care	Routinely‐collected data	ICD‐9 and10	2 130 000	89 554	CALIBER linked data from CPRD, MINAP, HES & ONS to identify newly recorded HF cases from 674 GP surgeries	Moderate
Mamas^76^	2017	2002–2011	UK	Primary care	Routinely collected data	Database HF code	1 750 000	56 658	Incident HF cases using Scottish Primary Care Clinical Informatics Unit data	Low
Pascual‐Figal^77^	2017	MUSIC 2003–2004 REDINSCOR 2007–2011	Spain	Cardiology outpatient	Prospective cohort	HF diagnostic criteria of local institutions	3446	3446	Data from MUSIC registry (8 specialist HF clinics with chronic symptomatic HF NYHA class II–III) and REDINSCOR registry (consecutive patients with HF NYHA class II–IV from 18 outpatient clinics)	Low
Taylor^11^	2017	1998–2012	UK	Primary care	Routinely collected data	Database codes based on NHS Clinical Terminology Browser and QOF guidelines	2 728 841	54 313	Incident HF cases in UK primary care from The Health Improvement Network (THIN)	Low
Sahle^78^	2017	1995–2001	Australia	Primary care	Prospective cohort	Defined as; 'significant dyspnoea with or without peripheral oedema together with definite physical signs of either left‐sided or congestive cardiac failure and/or the characteristic chest X‐ray appearance of left ventricular failure'	6083	145	Incident cases of HF within Australian National BP study – open‐label study of people with hypertension aged 65–84 years	Moderate
Stork^79^	2017	2009–2013	Germany	Cross‐discipline	Routinely collected data	ICD‐10‐GM	3 132 337	123 925	Patients with two HF‐related diagnoses within the German Health Risk Institute database	Moderate
Avula^80^	2018	2005–2012	USA	Cross‐discipline	Routinely collected data	ICD‐9 codes	28 914	28 914	Incident cases of HF among Kaiser Permanente Northern California healthcare members	Moderate
Eriksson^81^	2018	2001–2014	Sweden	Cross‐discipline	Registry	Individual clinician diagnosis	9654	9654	Incident HF cases in Swedish HF Registry, with LVEF ≥40%	Low

BNP, B‐type natriuretic peptide; BP, blood pressure; CPRD, Clinical Practice Research Datalink; COPD, chronic obstructive pulmonary disease; ESC, European Society of Cardiology; GP, general practice; HES, Hospital Episodes Statistics; HF, heart failure; HFpEF, heart failure with preserved ejection fraction; ICD‐9/10, International Classification of Diseases 9/10 (CM, GM refer to version used); LVDD, left ventricular end‐diastolic dimension; LVEF, left ventricular ejection fraction; LVSD, left ventricular systolic dysfunction; MINAP, Myocardial Ischaemia National Audit Project; NHS, National Health Service; NYHA, New York Heart Association; ONS, Office for National Statistics; QOF, Quality and Outcomes Framework; QUIPS, Quality in Prognosis Studies.

Demographic and baseline participant characteristics differed significantly between studies (online supplementary *Table* *S2*). Reporting of this information was inconsistent with ethnicity and deprivation indices only rarely included. However, co‐morbid cardiovascular disease was common, with hypertension the most frequent co‐morbidity, followed by diabetes and ischaemic heart disease. Treatment rates of key HF medications including angiotensin‐converting enzyme inhibitors/angiotensin receptor blockers, beta‐blockers and mineralocorticoid receptor antagonists improved over time. Some recent studies reported treatment rates close to 90%. Detailed prescribing information was lacking, meaning it was not possible to determine how many participants were treated with optimum dosage or the recommended combination of all three agents.

### Summary survival rates and causes of death

**Figure 1:**
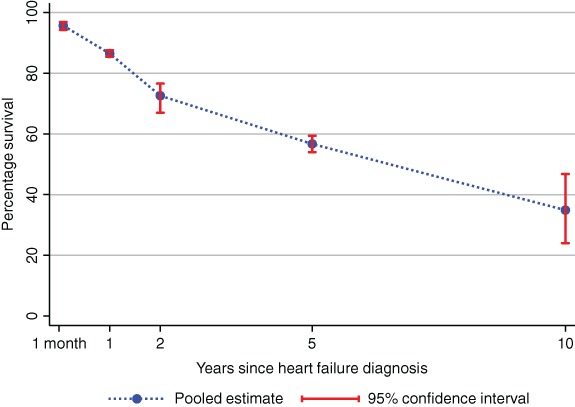
Combined survival rates for people with heart failure over time.

The pooled survival rates at 1 month, and 1, 2, 5 and 10 years, respectively, were 95.7% (95% confidence interval 94.3–96.9), 86.5% (85.4–87.6), 72.6% (67.0–76.6), 56.7% (54.0–59.4) and 34.9% (24.0–46.8)(*Figure* 1; online supplementary *Figures* *[Link]*, *[Link]*, *[Link]*, *[Link]*, *[Link]*). Only 19 studies reported data on cause of death, but in 14 of these a cardiovascular cause accounted for over 50% of the total deaths (*Table* 2).^25^, ^26^, ^34^, ^45^, ^47^, ^50^, ^51^, ^52^, ^53^, ^56^, ^60^, ^61^, ^63^, ^64^, ^67^, ^69^, ^72^, ^73^, ^74^, ^77^ HF tended to be the most frequent cause of death but there was significant variation in the reported proportion of deaths related directly to HF, ranging from 8% to 64%.

**Table 2:** Causes of mortality reported in included studies

First author	Year	Study subgroup	Cardiovascular mortality	Subgroups of cardiovascular mortality				Non‐cardiac mortality	Subgroups of non‐cardiovascular mortality				Unknown cause
				HF	Stroke	Sudden cardiac death	Coronary heart disease (including MI)		Pulmonary disease	Cancer	GI or GU disease	Other	
Cleland^26^	1987	Overall		8	1.3	75^a^	8			1.3			
Tsutsui^45^	2007	Overall	36					32					32
Henkel^50^	2008	Overall	57				36	43	12	10.8	5.2	5.2 (CNS disease)	
		HFpEF	51				29	49	14.2	11.3	5.4	6.9 (CNS disease)	
		HFrEF	64				43	36	10.1	9.7	5	3.6 (CNS disease)	
Crespo‐Leiro^69^	2016	Overall	49.8					23.2					27
Dokainish^72^	2017	Overall	46					16					38
Gupta^63^	2013	HFpEF	56				44						
		HFrEF	74				26						
Fragasso^61^	2013	Overall	63	24.6	7.6	15.8	13.9	37		16.3	5.6		
James^67^	2015	Overall	52.4	22.6	Cardiovascular non‐HF 29.8				20.2	9.5		6	11.9
		HFrEF	58.5	26.8	Cardiovascular non‐HF 31.7				17.1	12.2		2.4	9.8
		HFpEF	46.5	18.6	Cardiovascular non‐HF 27.9				23.3	7		9.3	14
Maggioni^64^	2013	Across regions	54.5			22		16.3					29.2
Pons^56^	2010	Overall	65.5	32.2	2.6	16	8.3	26.8	9.6	39.4	11.7	25.5 (sepsis)	7.7
Muntwyler^34^	2002	Overall	79										
Castillo^51^	2009	Total	95	64		24^a^	7	5					
Goda^52^	2009	Overall	85	47.5		22.5	15	15					
Hobbs^47^	2007	HF, no LVSD	44.8	17.2 definite, 23 probable ±	8	1.1^a^	13.8	55.2	23	14.9	3.4	5.7 (renal)	
		HF and LVSD	74	38.5 definite, 12.5 probable ±	7.7	3.8^a^	25	26	10.6	6.7	1	1.9 (renal)	
Taylor^60^	2012	HF, LVSD	72	32.1 definite ±			22.6	28	13.7	7.1			
		HF, no LVSD	48.4	19 definite ±			12	51.6	21.2	13			
Parashar	2009	White women	51.9										
		African‐American women	57.9										
		White men	56										
		African‐American men	45.4										
Singh^25^	2014	LVSD	69	33.1	9.8		20.2	31	8.6	14.7			
		HFpEF	43	15.3	13.6		13.6	57	13.6	21.2			
Farre^73^, ^74^	2017	Overall	46.2	27.1		7.5		29.6					24.2
		HFrEF	48.1	26.3		9.9		25.9					25.9
		HFmrEF	45.2	26.2		5.9		32.6					22.2
		HFpEF	42.3	29.5		2.7		36.7					20.9
Pascual‐Figal^77^	2017	HFrEF	80	49.7		24.5		20					
		HFmrEF	72.7	42.2		22.7		27.3					
		HFpEF	61.8	39.3		13.5		38.2					

Only studies reporting cause of mortality included. Blank cells indicate data were not reported in the original study. All figures refer to proportion of total mortality within the study. Selected subgroups of both cardiovascular and non‐cardiovascular mortality were reported in some studies, meaning in some cases the sum of the subgroup results are not equal to the combined mortality result.

CNS, central nervous system; GI, gastrointestinal; GU, genitourinary; HF, heart failure; HFmrEF, heart failure with mid‐range ejection fraction; HFpEF, heart failure with preserved ejection fraction; HFrEF, heart failure with reduced ejection fraction; LVSD, left ventricular systolic dysfunction; MI, myocardial infarction.

± HF cases recorded as either 'definite' or 'probable'. In Taylor, 'probable' HF mortality results not reported.

a Not specified that all cases of sudden death attributable to cardiac causes.

### Sensitivity analysis

The majority of studies were rated at low (*n* = 26) or moderate (n = 27) overall risk of bias (*Table* *S3*). Excluding the studies at moderate or high risk of bias in a sensitivity analysis did not alter the results. The pooled survival rate at 1 year across the remaining studies was 85.9% (84.1–87.7) and at 5 years 56.9% (52.1–61.7). The GRADE assessment suggests there is 'high' certainty in the summary findings (*Table* *S4*).

### Subgroup analysis by age

**Figure 2:**
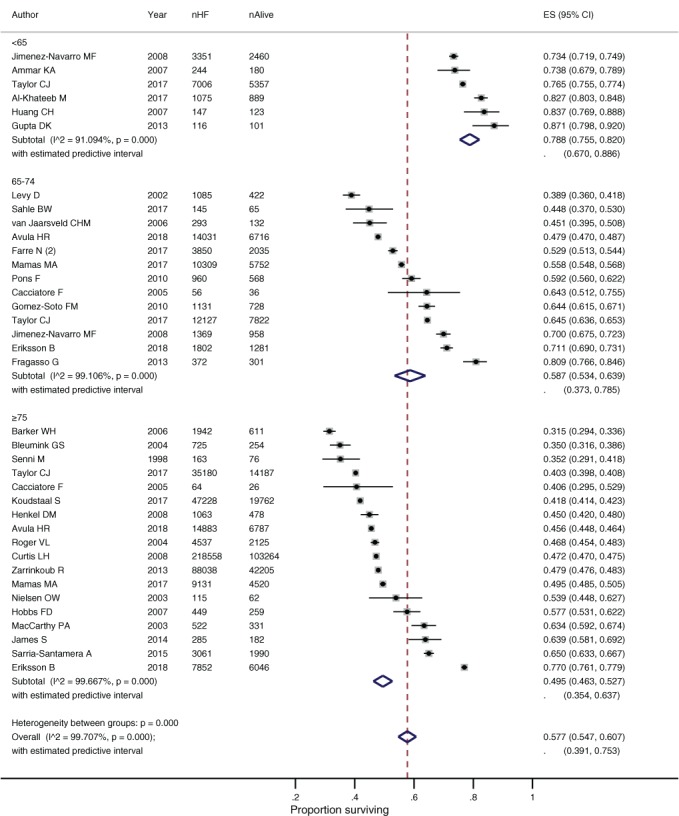
Survival of people with heart failure (HF) at 5 years by age at diagnosis. CI, confidence interval; ES, effect size.

Evidence from the forest plots and meta‐regression suggests survival rates decreased with increasing age at diagnosis (1‐year survival: R^2^ = 15.6%, *P*_trend_ = 0.005; 5‐year survival: R^2^ = 42.6%, *P*_trend_ < 0.001). Pooled survival rates at 1 year for people aged <65 years were 91.5% (88.2–94.3) compared to 83.3% (81.8–84.9) for people aged ≥ 75 years. By 5 years the respective survival rates were 78.8% (75.5–82.0) and 49.5% (46.3–52.7) (*Figure* 2; online supplementary *Table* *S5*).

The trend towards a worse prognosis in relation to age at diagnosis was also reported within individual studies.^11^, ^41^ In a recent analysis of survival rates within the UK THIN database, 5‐year survival rates were 50% amongst participants aged 75–84 years, compared to 81% amongst the youngest participants aged 45–54 years.^11^ In both cases, survival rates were significantly worse than for age‐matched participants of 72% and 98%, respectively.^11^

### Subgroup analysis by study setting

**Figure 3:**
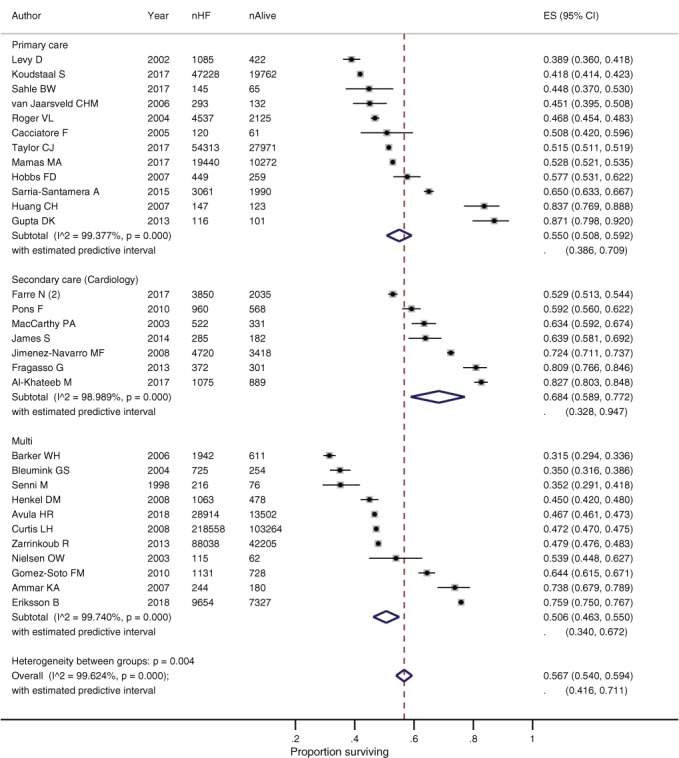
Survival of people with heart failure (HF) at 5 years by study setting. CI, confidence interval; ES, effect size.

The pooled 1‐ and 5‐year survival rates were significantly better for participants in secondary care studies compared to cross‐discipline studies (*Figure* 3). There was some evidence of improved survival in secondary care studies compared to primary care, with around 5% more participants alive at 1 year and 10% more at 5 years. The association between survival and setting was confirmed by meta‐regression (online supplementary *Table* *S5*). Individual secondary care studies with the poorest survival rates were those that purposively recruited either elderly frail participants, or those with a significant reduction in LVEF.^30^, ^31^ The primary care studies reporting the best survival rates used screening to detect incident HF cases.^48^, ^63^ Rates of key HF medication prescribing were consistently better in secondary care.

The four studies^36,45,48,52^ conducted in South‐East Asia reported better survival rates compared to Europe and North America, despite recruiting participants of comparable age and co‐morbid disease burden. One of these studies^48^ used screening to detect incident cases and the proportion of participants prescribed HF medication was also relatively high, which may explain this survival difference.

### Subgroup analysis by left ventricular ejection fraction

**Figure 4:**
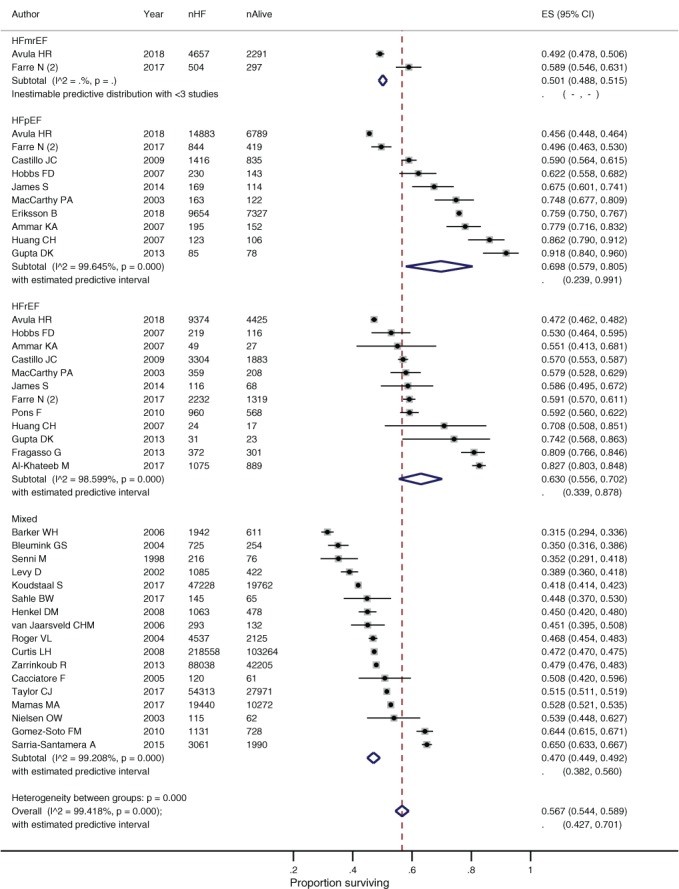
Survival of people with heart failure (HF) at 5 years by left ventricular ejection fraction. CI, confidence interval; ES, effect size.

The pooled survival rate at 5 years was better for patients with HFrEF than mixed ejection fraction (*Figure* 4). There was no significant difference in the pooled survival rates for HFpEF compared to HFrEF at either 1 or 5 years (online supplementary *Table* *S5*). A number of studies compared the risk of death by LVEF in their individual populations and found a preserved ejection fraction was associated with improved survival. Survival analysis from a community‐based screened cohort found patients with a LVEF <40% compared to LVEF >50% had a 1.80 (1.55–2.10) times greater risk of death over the study period, when adjusted for key factors such as age and sex.^60^ Other studies found the risk of death to be even greater for those with HFrEF, with hazard ratio of 2.62 (1.45–4.75),^50^ and 3.72 (1.80–7.68) reported.^39^ In every study reporting cause of death data categorised by LVEF, the proportion of total mortality attributed to cardiovascular disease and HF‐related mortality was greater for people with HFrEF than HFpEF (*Table* 2).

### Change in survival rates over time

**Figure 5:**
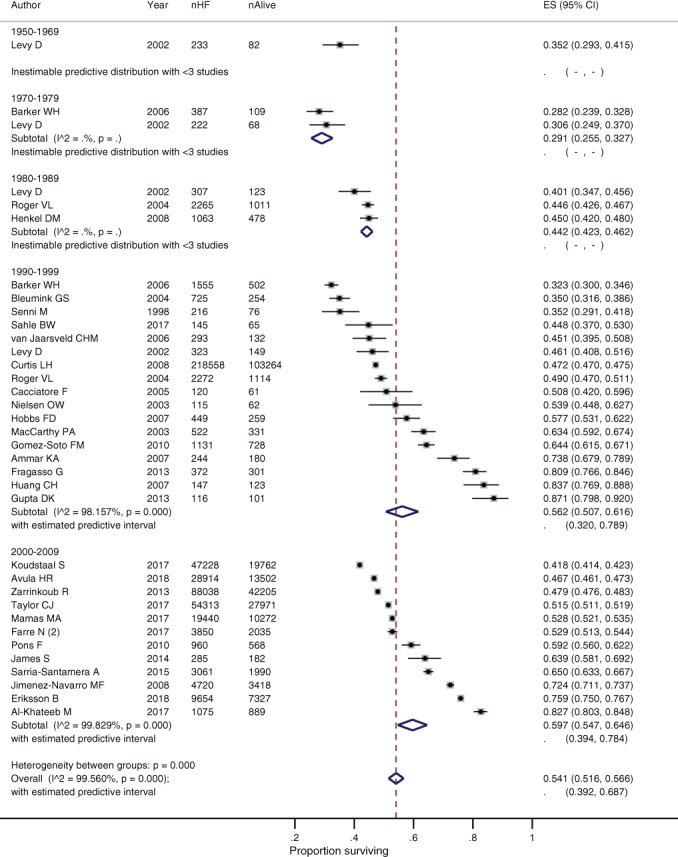
Survival of people with heart failure (HF) at 5 years by study decade. CI, confidence interval; ES, effect size.

Survival rates within each decade had high levels of heterogeneity (*Figure* 5), however over time and across the included studies there was a trend towards improvement in 1‐ and 5‐year survival rates (1‐year survival: R^2^ = 36.3%, *P*_trend_ < 0.001; 5‐year survival: R^2^ = 23.2%, *P*_trend_ = 0.013). Each decade since the 1970s has seen improving survival rates. The 1‐ and 5‐year pooled survival rates were 70.8% (64.7–76.3) and 35.2% (29.3–41.5) from the earliest reported time period, 1950–1969.^33^ By 2010–2019, 1‐ and 5‐year survival rates had reached 89.3% (84.3–93.4) and 59.7% (54.7–64.6).

Given the changes in treatment recommendations in the late 1990s, we conducted a subgroup analysis of pooled survival rates amongst all studies recruiting participants from the year 2000 onwards. The 1‐month, and 1‐, 2‐, and 5‐year survival rates for these groups were 95.2% (92.1–97.6), 89.3% (87.9–90.6), 78.9% (74.2–83.2) and 59.7% (54.7–64.6), respectively, slightly better than the overall pooled survival. Only one study reported 10‐year follow‐up data for participants post‐2000 with a survival rate of 29.5% (28.9–30.2).^11^

A number of studies have also demonstrated improving survival rates over time within their individual population. Framingham data show an improvement in 5‐year survival between 1950–1969 to 1990–1999 from 30% to 41% for men and from 43% to 55% for women.^33^ This trend is also seen in the Rochester Epidemiology Project.^40^ Recently, there have been more modest improvements in survival. A database study of over 400 000 people with HF in Ontario, found 1‐year mortality fell amongst outpatients with HF from 17.7% in 1997 to 16.2% in 2007.^59^ A study of 600 000 Medicare patients with incident HF reported a reduction in mortality from 67.5% to 64.9% for men and from 61.7% to 60.2% for women between 1994 and 2003.^49^

## Discussion

This is the first systematic review of prognosis in chronic HF and provides contemporary survival estimates applicable across high income countries. The analyses draw on survival data from 1.5 million people with chronic HF across 60 studies.

Survival rates have improved over time and 20% more people survive at both 1‐ and 5‐year follow‐up today compared to between 1950 and 1969. Survival rates improved sharply from the 1970s to 1990s, but there has been only a modest reduction in mortality in the past two decades. Increasing age at diagnosis is one key factor associated with a poor prognosis. Survival rates amongst people aged ≤ 65 years were almost 10% better at 1 year and over 30% better at 5 years, when compared to people aged ≥ 75 years. Survival rates were higher in studies recruiting participants from cardiology outpatient settings compared to cross‐discipline or primary care.

There was no significant difference in survival between HFrEF and HFpEF in our pooled analysis, though individual studies reported improved survival rates and lower rates of hospital admission and cardiovascular mortality for people with HFpEF. Both survival rates and prescribing of HF medication were significantly lower for patients where LVEF was not reported or analysed. This may be due to older trials with worse survival rates not reporting LVEF. It may also reflect certain populations, such as nursing home residents or older patients, are less likely to have LVEF measured despite having a worse prognosis. Nevertheless, recognising that patients who are not categorised by LVEF have a poorer outlook may have important implications for future assessment and treatment pathways.

The search strategy and eligibility criteria were designed to be inclusive, drawing studies from a wide range of geographical and healthcare settings. Source data from developing countries were less abundant but landmark cross‐continental studies provide data for these healthcare settings. Internationally, the lowest mortality rates were in South‐East Asian studies.

### Limitations

The diversity in study design and setting captured by the inclusive search strategy resulted in high levels of heterogeneity in each individual meta‐analysis. This included variations in participant characteristics that are likely to impact on prognosis. Screening was used to detect early HF in a small number of studies.^82^ Not all studies reported HF survival from time of diagnosis. Whilst primary care studies generally used routinely collected data sources to identify a first coded episode of HF, secondary care studies tended to calculate survival from first clinic visit, which may have been several years after diagnosis. Studies were categorised by setting to account for this potential time lag, though this was not apparent in our results. In practice, most patients with a confirmed diagnosis of HF will have input at some point from a cardiologist, except for some very frail patients who may be limited by cognitive or mobility issues. It is possible the differences seen in survival between settings reflect such variation in participant characteristics, though secondary care studies also reported higher rates of prescribing for key HF treatment. We plan to report more detail on prescribing rates in a separate paper. The definitions of cardiovascular and non‐cardiovascular death varied between studies as did the categories used in the cause of death subgroup analyses, making it difficult to compare these outcomes directly.

Outcome data are pooled from across a wide time period to capture changing survival rates over time. However, survival rates may not be directly comparable across these studies given there have been significant changes in HF management in the past 70 years, including the introduction of medications proven to improve prognosis for people with HFrEF. The statistical heterogeneity also reflects the large sample sizes of the included studies, which resulted in narrow confidence intervals. Even small differences in survival rates resulted in non‐overlapping confidence intervals and high I^2^ scores, a recognised limitation of this statistical measure in observational meta‐analysis.

The review included observational studies to present the real‐world outcomes for people with HF, outside of trial settings. Confounding is a recognised problem in these non‐randomised trials and reporting of important covariates was inconsistent. Missing data were a particular problem in earlier studies and those drawing on data from large primary care databases. Some meta‐regression results rely on data from a small number of studies, such as for HFmrEF and general secondary care clinics. However, similar results were observed when these small subgroups were combined with adjacent categories. Few studies reported echocardiogram findings or categorisation of HF by LVEF, despite the prognostic significance of this information. Accurate coding of HF is also a recognised limitation in routinely collected datasets.^83^, ^84^ However, this approach to epidemiological research is still felt to be valid and coding has been improving in line with performance payments and better access to diagnostic tests in primary care.^85^

### Comparison with existing literature

A recent European secondary care study reported 1‐year mortality rates for people with acute and chronic HF of 23% and 6%, respectively, compared to 3% for matched controls.^69^ In our pooled analysis, 1‐year mortality in chronic HF was above 10%. This may be because some people with a very poor prognosis are never admitted to hospital or referred to secondary care. Categorisation of HF has changed over time to recognise the importance of LVEF when considering treatment options and prognosis. Survival rates are better for people with HFpEF compared to HFrEF, once adjusted for key covariates including age, sex, and aetiology of HF.^86^ However, people with HFpEF are more likely to be older and have significant co‐morbid disease, meaning the unadjusted HFrEF and HFpEF survival rates are similar. This may explain why there was no significant difference in survival in our subgroup analysis based on LVEF.

### Research implications

Our results provide a reference source for clinicians, patients and policy makers, to inform population prognostic estimates. The subgroup analyses help to provide adjusted survival estimates based on key variables, such as age at time of diagnosis. Further work is needed to refine prognostic models for individuals with chronic HF. Existing tools, such as the Seattle Heart Failure Model and MAGGIC HF risk tool, lack specificity and sensitivity data that are applicable to clinical practice.^86^, ^87^ Reducing uncertainty and confusion about the outcomes in HF could lead to improvements in advanced care planning, treatment adherence and integration with wider healthcare teams such as palliative care.^16^, ^88^

Survival rates in HF remain poor despite modest improvements over time. Investment in healthcare infrastructure and public health initiatives for conditions with similar outcomes such as cancer and stroke have seen improvements in morbidity and mortality.^89^, ^90^ This review suggests that targeted allocation of resources towards improving early diagnosis, prescribing and treatment adherence and multi‐disciplinary models of care may lead to further reductions in mortality for people with HF.

## Conclusion

There have been modest improvements in survival rates for people with chronic HF over the past 70 years. Despite this, the 5‐year survival rate is close to 50% and many people will die directly from HF or from related cardiovascular disease. Older populations are at the greatest risk of death, presenting a looming challenge to healthcare systems given changing global demographics. Our results draw from very heterogeneous data sources and when applying survival estimates to any individual, consideration should be given to factors such as their age, co‐morbid disease, treatment, and LVEF. Further research is needed to develop the evidence base around key prognostic indicators for patients with chronic HF that will enable population estimates to be refined for individuals. Greater understanding and awareness of chronic HF survival rates can facilitate better multi‐disciplinary team working and inform advanced care planning between patients and healthcare professionals.

## Supporting information


**Methods S1.** MOOSE (Meta‐analyses Of Observational Studies in Epidemiology) checklist.

**Methods S2.** Risk of bias and quality assessment.

**Table S1.** Search strategy.

**Table S2.** Prevalence of co‐morbid disease, cardiovascular risk factors and heart failure medication across studies.

**Table S3.** Risk of bias assessment using the Quality in Prognosis Studies tool.

**Table S4.** GRADE risk of bias assessment across studies.

**Table S5.** Subgroup and meta‐regression analyses by age at diagnosis, setting, left ventricular ejection fraction, and date.

**Figure S1.** Survival of people with heart failure at 1 month.

**Figure S2.** Survival of people with heart failure at 1 year.

**Figure S3.** Survival of people with heart failure at 2 years.

**Figure S4.** Survival of people with heart failure at 5 years.

**Figure S5.** Survival of people with heart failure at 10 years.

**Figure S6.** PRISMA flow diagram of study selection.
