# Supplementary material for: Survival of patients with chronic heart failure in the community: a systematic review and meta‐analysis
Source: Eur J Heart Fail. 2019 Sep 16;21(11):1306–25. doi: 10.1002/ejhf.1594 (PMC6919428; doi:10.1002/ejhf.1594)
Supplement: Supplementary file 1 — Methods S1. MOOSE (Meta‐analyses Of Observational Studies in Epidemiology) checklist. [file EJHF-21-1306-s012.docx]

**Supplementary results 1. Risk of bias and quality assessment**

The majority of studies were rated at low (n=26) or moderate (n=27) overall risk of bias in the QUIPS assessment (eTable 4). Reliable outcome data on mortality was readily available across the studies. Most included large sample sizes and there were low attrition rates, which rarely impacted on the summary findings. HF classification was based on validated diagnostic criteria and international guidelines, which reduced the risk of inter-rater variability. Although the categorisation of HF has changed over time, history, examination and echocardiogram findings have formed the basis of assessment since the 1950s.

Common sources of bias reflected the individual study methodology. The large database studies relied on primary care codes from routinely collected data to capture incident cases and so were prone to misclassification bias. The prospective cohort studies relied on volunteer participants and so were at risk of selection bias. The majority of the seven studies rated at high risk of bias were earlier publications, perhaps reflecting changes in study reporting standards over time. They lacked key information, such as the approach to recruitment or baseline participant characteristics and so were deemed at high risk of bias in terms of ‘study participants’ or ‘confounding’.

The GRADE assessment suggests there is ‘high’ certainty in the summary findings (eTable 5). The results are rated as ‘important’, given their clinical relevance to a large global population of people with HF. Risk of bias was deemed to be ‘not serious’ based on the QUIPS assessment and the fact results were consistent in a sensitivity analysis excluding those studies at high risk of bias. The large amount of included survival data meant we were able to report precise summary estimates. Inconsistency was rated as ‘serious’ given the variation in survival estimates, non-overlapping confidence intervals and high I^2^ score. Indirectness was also rated as ‘serious’ given the variation in studies in terms of healthcare setting and recruitment dates.
