# Supplementary material for: Survival of patients with chronic heart failure in the community: a systematic review and meta‐analysis
Source: Eur J Heart Fail. 2019 Sep 16;21(11):1306–25. doi: 10.1002/ejhf.1594 (PMC6919428; doi:10.1002/ejhf.1594)
Supplement: Supplementary file 3 — Table S1. Search strategy. [file EJHF-21-1306-s001.docx]

**Supplementary table 1. Search strategy**

We searched the following databases from inception to August 2018: MEDLINE, Embase, CINAHL, the Database of Abstracts of Reviews of Effects and the Clinical Trials Register. PROSPERO and the Trip Database were checked for ongoing or recently completed systematic reviews.

An initial validation search was run for specificity and sensitivity, following which the terms ‘general population’ and ‘epidemiol*’ were added, based on feedback from the departmental librarian and a primary care systematic reviewer.

**MEDLINE search strategy**

| 1 | heart failure.ti. or *heart failure/ |
| --- | --- |
| 2 | cardiac failure.ti. or *heart failure/ |
| 3 | congestive cardiac failure.mp. or *congestive heart failure/ |
| 4 | 1 or 2 or 3 |
| 5 | mortality/ or cardiovascular mortality/ or mortality.mp. |
| 6 | death/ or death.mp. |
| 7 | survival.mp. or median survival time/ or long term survival/ or overall survival/ or survival/ |
| 8 | outlook.mp. |
| 9 | prognosis/ or prognosis.mp. |
| 10 | 5 or 6 or 7 or 8 or 9 |
| 11 | Clinical study/ |
| 12 | Longitudinal study/ |
| 13 | Retrospective study/ |
| 14 | Prospective study/ |
| 15 | Randomized controlled trials/ |
| 16 | Cohort analysis/ |
| 17 | (Cohort adj (study or studies)).mp. |
| 18 | (follow up adj (study or studies)).tw. |
| 19 | (observational adj (study or studies)).tw. |
| 20 | (epidemiologic$ adj (study or studies)).tw. |
| 21 | survival analysis/ |
| 22 | 14 not 15 |
| 23 | or/11-13,16-22 |
| 24 | stable.mp. |
| 25 | chronic.mp. |
| 26 | community/ or community.mp. |
| 27 | primary care.mp. or primary medical care/ |
| 28 | general practice.mp. or general practice/ |
| 29 | family practice.mp. or general practice/ |
| 30 | general population.mp. or population/ |
| 31 | epidemiolog*.mp. |
| 32 | 24 or 25 or 26 or 27 or 28 or 29 or 30 or 31 |
| 33 | 4 and 10 and 23 and 32 |
| 34 | limit 33 to ("all adult (19 plus years)" and humans) |
