# Supplementary material for: Survival of patients with chronic heart failure in the community: a systematic review and meta‐analysis
Source: Eur J Heart Fail. 2019 Sep 16;21(11):1306–25. doi: 10.1002/ejhf.1594 (PMC6919428; doi:10.1002/ejhf.1594)
Supplement: Supplementary file 4 — Table S2. Prevalence of co‐morbid disease, cardiovascular risk factors and heart failure medication across studies. [file EJHF-21-1306-s002.docx]

**Supplementary table 2. Prevalence of comorbid disease, cardiovascular risk factors and heart failure medication across studies**

| First author | Study subgroups (where not reported for overall cohort ) | Mean age (years) | Smokers (ever unless stated) | Overweight and obese or mean BMI | Female | Diabetes | IHD | MI | Hyper-tension | Stroke | AF | Cancer | COPD |
| --- | --- | --- | --- | --- | --- | --- | --- | --- | --- | --- | --- | --- | --- |
| Akwo EA |  | 55.5 | 65.4 | 44.8 | 62.6 | 26.5 |  | 8.6 | 62.5 | 9.6 |  |  |  |
| Al-Khateeb M |  | 57.3 | 28.8 | 36.2 | 28 | 74.6 |  |  | 64 |  |  |  |  |
| Barker WH | 1970-74 (female) | All > 65 |  |  | 100 | 15 | 21 | 32 | 82 |  | 31 | 6 | 2 |
|  | 1970-74 (male) | All > 65 |  |  | 0 | 18 | 30 | 39 | 70 |  | 27 | 11 | 12 |
|  | 1990-94 (female) | All > 65 |  |  | 100 | 24 | 22 | 28 | 83 |  | 26 | 13 | 17 |
|  | 1990 - 94 (male) | All > 65 |  |  | 0 | 22 | 25 | 33 | 79 |  | 29 | 21 | 20 |
| Koseki Y | Dilated cardiomyopathy | 82 |  |  | 27.3 |  |  |  |  |  |  |  |  |
|  | Myocardial infarction | 70 |  |  | 24.5 |  |  |  |  |  |  |  |  |
|  | Valvular heart disease | 72 |  |  | 39.6 |  |  |  |  |  |  |  |  |
|  | Left ventricular hypertrophy | 71 |  |  | 37 |  |  |  |  |  |  |  |  |
| Tsutsui H |  | 74 |  |  | 54 |  | 30 |  | 35 |  | 40 |  |  |
| Grundtvig M |  | 70.8 | 16* | BMI 26 | 30 | 19 | 57 |  | 30 | 11 | 27 |  | 17 |
| Cicoira M |  | 76.7 |  |  | 29 |  | 66 |  |  |  | 29 |  |  |
| Senni M (2005) |  | 64 |  | 12 | 23 |  | 45 |  |  |  |  |  |  |
| Henkel DM |  | 76.4 | 53 | BMI 27.1 | 54 | 19 | 35 | 23 | 68 |  |  | 18 | 22 |
| Chen HH |  | 79 |  |  | 76 | 18 | 13 | 18 | 72 |  | 29 |  |  |
| Ammar KA | Stage 0 |  |  | 0 | 53 | 0 | 0 | 0 | 0 |  |  |  |  |
|  | Stage A |  |  | 68 | 48 | 12 | 9 | 0 | 43 |  |  |  |  |
|  | Stage B |  |  | 34 | 50 | 9 | 17 | 8 | 37 |  |  |  |  |
|  | Stage C |  |  | 47 | 63 | 15 | 35 | 18 | 58 |  |  |  |  |
|  | Stage C1 |  |  | 48 | 68 | 13 | 26 | 12 | 56 |  |  |  |  |
|  | Stage C2 |  |  | 43 | 43 | 27 | 75 | 43 | 63 |  |  |  |  |
|  | Stage D |  |  | 60 | 40 | 80 | 75 | 80 | 80 |  |  |  |  |
| Crespo-Leiro MG |  | 64.9 | 11.2* | BMI 28.1 | 28.8 | 31.9 | 43.1 |  | 58.3 | 9.5 | 37.7 |  | 13.9 |
| Cacciatore F | No HF | 74.0 |  |  | 56.4 |  | 10 |  |  |  |  |  |  |
|  | HF | 75.9 |  |  | 60 |  | 56.7 |  |  |  |  |  |  |
| Dokainish H |  | 59 | 6* |  | 39 | 29 | 39 |  |  |  |  |  | 6 |
| Gupta DK | No HF | 58.5 | 20* | 45 | 63 | 22 | 4 |  | 59 | 2 |  |  |  |
|  | HFpEF | 61.1 | 12* | 71 | 85 | 42 | 13 |  | 85 | 5 |  |  |  |
|  | HFrEF | 62 | 13* | 61 | 65 | 68 | 32 |  | 84 | 16 |  |  |  |
| Huang CH | No CHF | 54.2 |  |  | 54 | 12.5 | 4.9 |  | 28.4 |  | 0.8 |  |  |
|  | HFpEF | 58.1 |  |  | 55.1 | 15.4 | 8.3 |  | 39.8 |  | 4.1 |  |  |
|  | HFrEF | 62.1 |  |  | 47.9 | 12.5 | 2.5 |  | 45.8 |  | 8.3 |  |  |
| Fragasso G |  | 66 |  |  | 27.7 | 31 | 42 | 45 | 63 |  | 25 |  |  |
| Gomez-Soto FM | Year 2000 | 67.3 | 36 | 35.4 | 52.6 | 25.8 | 45.7 |  | 44 | 15.8 |  | 12.2 | 36.8 |
|  | Year 2003 | 69.6 | 33.6 | 36.6 | 52.4 | 31.9 | 46.5 |  | 46.1 | 16.1 |  | 12.5 | 36.9 |
|  | Year 2006 | 71.7 | 31.2 | 38.2 | 53 | 36.8 | 46.2 |  | 47.8 | 16.4 |  | 12.7 | 37.5 |
| Curtis LH | 1994 | 78.4 |  |  | 58.2 | 27.7 | 62.6 |  | 64.5 | 25.9 |  | 4.7 | 39.1 |
|  | 1995 | 78.4 |  |  | 58.7 | 28.2 | 62.6 |  | 67 | 27.3 |  | 4.7 | 39.4 |
|  | 1996 | 78.4 |  |  | 58.6 | 28.6 | 63 |  | 69.6 | 28.1 |  | 4.8 | 40.4 |
|  | 1997 | 78.4 |  |  | 58.2 | 60.4* | 63.3 |  | 72.3 | 29.3 |  | 4.9 | 41.4 |
|  | 1998 | 78.4 |  |  | 58.1 | 31.4 | 63.5 |  | 74.2 | 29.7 |  | 4.8 | 41.7 |
|  | 1999 | 78.5 |  |  | 58.8 | 32.3 | 63.4 |  | 76.3 | 30.4 |  | 4.5 | 42.4 |
|  | 2000 | 78.4 |  |  | 58.1 | 33.9 | 63.7 |  | 78.3 | 30.5 |  | 4.4 | 42.7 |
|  | 2001 | 78.3 |  |  | 57.4 | 35.1 | 63.5 |  | 80.6 | 31.2 |  | 4.5 | 43.8 |
|  | 2002 | 78.4 |  |  | 57.9 | 36.1 | 64.2 |  | 82.9 | 31.7 |  | 4.4 | 45.3 |
|  | 2003 | 78.4 |  |  | 57.1 | 37.4 | 64.4 |  | 84.5 | 32.3 |  | 4.5 | 46.3 |
| Ansari M | HFpEF | 69.4 |  | 48 | 50 | 27 | 44 | 25 | 75 | 18 |  |  | 30 |
|  | HFrEF | 66.3 |  | 30 | 30 | 25 | 60 | 40 | 67 | 13 |  |  | 21 |
| Jimenez-Navarro MF | Male | 64 |  |  | 71 | 29 | 50 | 41 | 35 |  |  |  |  |
|  | Female | 70 |  |  | 29 | 39 | 30 | 19 | 50 |  |  |  |  |
| McAlister FA | HFrEF | 65 |  |  | 28 | 20 | 70 | 56 | 24 |  |  |  |  |
|  | HFpEF | 69 |  |  | 50 | 21 | 43 | 31 | 40 |  |  |  |  |
| van Jaarsveld CHM | No HF | 69 |  |  | 56 | 7 | 19 |  | 22 | 2 |  |  |  |
|  | HF | 75.5 |  |  | 52 | 14 | 40 |  | 29 | 4 |  |  |  |
| Koudstaal S | CPRD | 78.8 | 46.7 | BMI 27.1 | 51.3 | 8.9 | 43.9 | 21.1 | 74.9 | 2.8 | 27.6 | 19.6 | 17.5 |
|  | CPRD and HES | 79.8 | 48.6 | BMI 27.3 | 49.7 | 16.4 | 56.7 | 31.7 | 84 | 4.4 | 45.6 | 19.3 | 23 |
|  | HES | 80.4 | 49.2 | BMI 27 | 52.2 | 17.9 | 50.1 | 28.9 | 80.3 | 6.4 | 40.6 | 23.4 | 21 |
| James S | HFrEF | 77.7 |  | BMI 28.1 | 38.8 | 12.9 | 47.4 |  | 49.1 | 7.8 | 44.8 | 14.7 | 19.8 |
|  | HFpEF | 80.1 |  | BMI 30 | 56.8 | 18.9 | 24.9 |  | 70.4 | 8.3 | 59.8 | 10.7 | 13.6 |
| Levy D | 1950-1969 | 62.7 |  |  | 51 |  |  |  |  |  |  |  |  |
|  | 1970-1979 |  |  |  |  |  |  |  |  |  |  |  |  |
|  | 1980-1989 |  |  |  |  |  |  |  |  |  |  |  |  |
|  | 1990-1999 | 80.0 |  |  |  |  |  |  |  |  |  |  |  |
| Maggioni AP |  | 66 |  | BMI 28 | 29.7 | 29 | 40.5 |  | 58.3 | 10.5 | 38.6 |  | 15.1 |
| Mamas MA | Male | 70.5 | 59.6 |  | 0 | 21.7 | 61.1 | 43.1 | 45.7 | 7.3 (stroke or TIA) | 5.4 | 2.2 | 16.6 |
|  | Female | 76.4 | 39 |  | 100 | 18.7 | 47.8 | 29.2 | 54.6 | 15.4 | 26 | 4 | 15.9 |
| Pons F |  | 69 |  | BMI 27.1 | 29.1 | 39.3 | 55.4 |  | 58.6 |  | 16.9 |  | 20.8 |
| Cleland JGF |  | 59 |  |  | 16 |  | 61.2 |  |  |  |  |  |  |
| Muntwyler J |  | 75 |  |  | 44 | 18 | 39 |  | 20 |  | 31 |  |  |
| Castillo JC | HFpEF | 71 |  |  | 53 | 36 | 32 | 18 | 66 |  | 46 |  |  |
|  | HFrEF | 64 |  |  | 28 | 31 | 47 | 40 | 49 |  | 30 |  |  |
| Goda A | Male | 73 |  |  | 0 | 6.8 | 51.7 |  |  |  |  |  |  |
| Bleumink GS | Male | 82.5 |  |  | 0 |  |  |  |  |  |  |  |  |
|  | Female | 77.5 |  |  | 100 |  |  |  |  |  |  |  |  |
| Hobbs FD | HF, no LVSD | 75.5 |  | BMI 26 | 52.2 | 11.3 | 33.9 | 20.4 | 44.4 |  |  |  |  |
|  | HF and LVSD | 70.5 |  | BMI 27.6 | 30.6 | 19.6 | 51.6 | 54.8 | 40.6 |  |  |  |  |
|  | No HF, LVSD | 70.2 | 74.3 | BMI 26.3 | 16.5 | 7.3 | 35.8 | 62.3 | 37.6 |  |  |  |  |
|  | No HF or LVSD | 63.3 | 58.5 | BMI 26.7 | 51.3 | 7.5 | 14.7 | 9.7 | 36.1 |  |  |  |  |
| Yeung DF | Outpatients | 76 |  |  | 50.3 | 13.8 | 25.5 |  | 23.9 | 9.1 | 12.1 | 7.3 | 11.6 |
| Taylor CJ (2012) | HF |  | 21.5* | BMI 27.9 | 48.5 | 19.8 | 42.9 IHD; 23.1 Angina | 22.6 | 53.1 | 9.2 | 28.5 |  |  |
|  | No HF |  | 22.9* | BMI 26.4 | 48.8 | 10.8 | 17.2 | 6.4 | 41.9 | 5.9 | 7.1 |  |  |
| Farre N (1) |  | 77.4 |  |  | 54.8 | 42.2 | 47.9 | 15 | 97.3 | 18.3 | 47.6 | 21 | 32.4 |
| Stork S | Total (includes below subgroup) | 76.2 |  |  | 55 |  |  |  |  |  |  |  |  |
|  | Newly diagnosed in 2011 |  |  | 21.8 | 50.9 | 49.8 | 44.4 |  | 81.7 |  | 29.7 |  | 18.2 |
| Sarria-Santamera A |  | 76.47 |  | 22.8 | 62.5 | 26.1 | 14.7 |  | 67 | 6 | 27 |  |  |
| Stalhammar J |  | 77.1 |  | 59.6 | 59.8 | 20.4 | 21.1 | 23.3 | 55.4 | 8 | 37.9 |  | 13.8 |
| Frigola-Capell E |  | 77 |  |  | 60 | 29.8 | 25.8 |  | 70.6 |  |  |  | 14.6 |
| Taylor CJ (2017) | No HF, no LVSD | 63.3 | 58.5 | BMI 26.7 | 51.3 | 7.5 | 14.7 | 9.7 | 36.1 |  |  |  |  |
|  | HF, no LVSD | 75.5 | 62.2 | 26 | 52.2 | 11.3 | 33.9 | 20.4 | 44.4 |  |  |  |  |
|  | No HF, LVSD | 70.2 | 74.3 | 26.3 | 16.5 | 7.3 | 35.8 | 62.3 | 37.6 |  |  |  |  |
|  | HF, LVSD | 70.5 | 69.4 | 27.6 | 30.6 | 19.6 | 51.6 | 54.8 | 40.6 |  |  |  |  |
| Devroey D |  | 79 |  |  | 56 |  |  |  |  |  |  |  |  |
| Parashar S | White women | 82 | 42 | BMI 26.8 | 100 | 17 | 32 | 15 | 68 | 12 | 8 |  |  |
|  | African-American women | 81 | 40 | BMI 31.4 | 100 | 38 | 31 | 9 | 76 | 12 | 5 |  |  |
|  | White men | 81 | 68 | BMI 26.9 | 0 | 23 | 45 | 27 | 58 | 13 | 9 |  |  |
|  | African-American men | 79 | 71 | BMI 27.1 | 0 | 29 | 30 | 21 | 74 | 12 | 6 |  |  |
| Raymond I |  | 66 |  |  | 57 | 6.3 | 4.6 |  | 23.2 |  | 3.2 |  | 10 |
| Roger VL | Male total | 73 | 68 | 63 | 0 | 29 | 32 | 26 | 61 |  |  |  |  |
|  | Female total | 79 | 35 | 49 | 100 | 19 | 24 | 19 | 72 |  |  |  |  |
| MacCarthy PA | HFpEF | 62.5 |  |  | 28 |  | 76 | 65 | 6 |  |  |  |  |
|  | HFrEF | 62.3 |  |  | 20 |  | 79 | 66 | 3 |  |  |  |  |
| Nielsen OW | Hospital HF |  |  | 76.7 | 32 |  | 45 | 53 | 32 | 37 |  |  |  |
|  | GP HF | 75.8 |  |  | 66 |  | 10 | 6 | 47 |  | 27 |  |  |
|  | Non HF | 74.4 |  |  | 65 |  | 22 | 12 | 46 |  | 24 |  |  |
|  | Control | 74.1 |  |  | 61 |  | 0 | 0 | 0 |  | 0 |  |  |
| Mosterd A | No HF | 68.6 | 23.1* | 61.9 | 59.2 | 10.3 | 6 | 9.7 | 30.2 |  | 1.8 |  |  |
|  | HF | 77.3 | 22.1* | 62 | 60.2 | 17.5 | 37.2 | 49.2 | 33.8 |  | 21.4 |  |  |
| Niebauer J | LVEF 11-20% | 58.4 |  |  |  |  |  |  |  |  |  |  |  |
|  | LVEF </=10% | 56.0 |  |  |  |  |  |  |  |  |  |  |  |
| Senni M (1998) | Combined | 77.3 |  |  | 42 |  | 40 | 29 | 52 |  | 24 |  | 23 |
| Ho KKL | Combined | 70 | 33 | BMI 27.2 | 49 | 19 |  |  | 74 |  |  |  |  |
| Singh R | LVSD | 74 | 69 |  | 39 | 16 | 52 |  | 43 | 13 | 25 |  | 22 |
|  | HFpEF | 78 | 51.4 |  | 65 | 23 | 42 |  | 84 | 4 | 36 |  | 5 |
|  | Non HF (Other) | 75 | 51 |  | 65 | 12 | 26 |  | 49 | No data | 14 |  | 28 |
| Avula HR |  | 72.8 |  |  | 45 | 41.6 |  | 14.2 | 80.1 | 6.2 | 31.9 | 9.1 | 36.7 |
| Eriksson B | Primary care | 77.5 | 43 |  | 46.7 | 21.1 | 57.8 |  | 67 |  | 53 |  | 24.5 |
|  | Hospital care | 70.3 | 42.4 |  | 36.3 | 20.1 | 32.7 |  | 48.9 |  | 47.2 |  | 15.2 |
| Farre N (2) |  | 68.2 |  | BMI 27.8 | 67 | 43.2 | 44.7 |  | 69.4 |  | 27.9 |  | 20.1 |
| Pascual-Figal DA | HFrEF | 64.4 | 17.3* | BMI 28.1 | 23.2 | 39.7 | 51.4 | 42.5 | 60.5 |  | 18.8 |  |  |
|  | HFmrEF | 66.7 | 10.3* | BMI 29.3 | 27 | 45.9 | 55.7 | 44.6 | 66.3 |  | 20.4 |  |  |
|  | HFpEf | 72.1 | 8* | BMI 30.1 | 57.2 | 40.6 | 26 | 22.9 | 80.6 |  | 34.8 |  |  |
| Sahle BW | HF | 75 | 69 | 70 | 36 | 18 | 21 |  |  |  |  |  |  |
|  | Non-HF | 72 | 52 | 69 | 52 | 7 | 11 |  |  |  |  |  |  |
| Zarrinkoub R | Women | 81 |  |  |  | 27 | 54 |  | 67 | 28 | 49 |  | 22 |
|  | Men | 74 |  |  |  | 33 | 64 |  | 65 | 28 | 54 |  | 23 |

AF – atrial fibrillation; BMI – body mass index; COPD – chronic obstructive pulmonary disease; CPRD – Clinical Practice Research Datalink; GP – General Practice; HES – Hospital episode statistics; HF – heart failure; HFmrEF – heart failure with mid-range ejection fraction; HFpEF – heart failure with preserved ejection fraction; HFrEF – heart failure with reduced ejection fraction; IHD – ischaemic heart disease; LVEF – left ventricular ejection fraction; LVSD – left ventricular systolic dysfunction; MRA – mineralocorticoid receptor antagonist

*- current smokers
