# Supplementary material for: Survival of patients with chronic heart failure in the community: a systematic review and meta‐analysis
Source: Eur J Heart Fail. 2019 Sep 16;21(11):1306–25. doi: 10.1002/ejhf.1594 (PMC6919428; doi:10.1002/ejhf.1594)
Supplement: Supplementary file 5 — Table S3. Risk of bias assessment using the Quality in Prognosis Studies tool. [file EJHF-21-1306-s003.docx]

**Supplementary table 3. Risk of bias assessment using the Quality in Prognosis Studies tool**

| First Author | Publication year | Study participation | Study attrition | Prognostic factor | Outcome measurement | Study confounding | Statistical analysis & reporting | Summary score |
| --- | --- | --- | --- | --- | --- | --- | --- | --- |
| Cleland JGF^50^ | 1987 | High | Moderate | Moderate | Moderate | Moderate | Moderate | High |
| Ho KKL^51^ | 1993 | Low | Low | Low | Low | Moderate | Low | Moderate |
| Senni M^52^ | 1998 | Low | Low | Low | Low | Low | Low | Low |
| McAlister FA^53^ | 1999 | High | Low | Low | Low | Low | Low | Moderate |
| Niebauer J^28^ | 1999 | High | Low | Moderate | Low | Moderate | Low | High |
| Cicoira M^30^ | 2001 | High | Low | Low | Moderate | High | Low | High |
| Mosterd A^8^ | 2001 | Low | Low | Low | Low | Low | Low | Low |
| Chen HH^54^ | 2002 | Moderate | Moderate | Low | Low | Low | Low | Low |
| Levy D^36^ | 2002 | Moderate | Low | Low | Low | Moderate | Moderate | Moderate |
| Muntwyler J^55^ | 2002 | Moderate | Moderate | Low | Low | Moderate | Low | Moderate |
| Ansari M^26^ | 2003 | Moderate | Low | Moderate | Low | Low | Low | Moderate |
| Koseki Y^56^ | 2003 | Moderate | Moderate | Moderate | Low | High | Low | High |
| MacCarthy PA^57^ | 2003 | Moderate | Low | Moderate | Low | Moderate | Moderate | Moderate |
| Nielsen OW^58^ | 2003 | Moderate | Low | Moderate | Low | Low | Low | Moderate |
| Bleumink GS^1^ | 2004 | Low | Low | Low | Low | Moderate | Moderate | Moderate |
| Raymond I^35^ | 2004 | Low | Low | Low | Low | Low | Low | Low |
| Roger VL^37^ | 2004 | Low | Low | Low | Low | Moderate | Low | Low |
| Cacciatore F^29^ | 2005 | Moderate | Moderate | Moderate | Moderate | Moderate | Low | Moderate |
| Senni M^59^ | 2005 | Moderate | Moderate | Low | Low | Low | Low | Low |
| Barker WH^60^ | 2006 | Moderate | Low | Low | Low | Moderate | Moderate | Moderate |
| van Jaarsveld CHM^61^ | 2006 | Low | Moderate | Moderate | Low | Moderate | Low | Moderate |
| Tsutsui H^62^ | 2007 | Moderate | Low | Low | Low | Low | Low | Low |
| Ammar KA^63^ | 2007 | Moderate | Moderate | Low | Low | Moderate | Low | Moderate |
| Hobbs FD^64^ | 2007 | Low | Low | Low | Low | Low | Moderate | Low |
| Huang CH^31^ | 2007 | Low | Moderate | Low | Low | Moderate | Low | Moderate |
| Curtis LH^39^ | 2008 | Moderate | Low | Moderate | Low | Moderate | Low | Moderate |
| Henkel DM^34^ | 2008 | Low | Low | Low | Low | Moderate | Low | Low |
| Jimenez-Navarro MF^65^ | 2008 | Moderate | Moderate | Low | Moderate | Moderate | Low | Moderate |
| Castillo JC^66^ | 2009 | Moderate | Low | Low | Low | Low | Low | Low |
| Goda A^67^ | 2009 | Moderate | Low | Low | Low | Moderate | Low | Moderate |
| Parashar S^68^ | 2009 | Low | Low | Moderate | Low | Low | Low | Low |
| Devroey D^69^ | 2010 | Moderate | Low | Moderate | Low | Moderate | Moderate | High |
| Gomez-Soto FM^70^ | 2010 | Low | Moderate | Low | Low | Low | Low | Low |
| Grundtvig M^71^ | 2010 | Moderate | Moderate | Low | Low | Low | Low | Low |
| Pons F^72^ | 2010 | High | Low | Moderate | Low | Moderate | Low | High |
| Yeung DF^38^ | 2012 | Low | Low | Moderate | Low | Moderate | Low | Low |
| Taylor CJ^33^ | 2012 | Low | Low | Low | Low | Low | Low | Low |
| Fragasso G^73^ | 2013 | Moderate | Low | Moderate | Low | Moderate | Low | Moderate |
| Frigola-Capell E^74^ | 2013 | Low | Low | Moderate | Low | Low | Low | Low |
| Gupta DK^32^ | 2013 | Moderate | Low | Low | Low | Low | Low | Low |
| Maggioni AP^75^ | 2013 | Moderate | Low | Moderate | Low | Moderate | Low | Moderate |
| Zarrinkoub R^76^ | 2013 | Low | Low | Moderate | Low | Moderate | Low | Low |
| James S^77^ | 2014 | Moderate | Moderate | Low | Low | Moderate | Low | Moderate |
| Singh R^78^ | 2014 | Moderate | Low | Low | Low | Low | Low | Moderate |
| Stalhammar J^79^ | 2014 | Moderate | Moderate | Moderate | Low | Moderate | Moderate | Moderate |
| Sarria-Santamera A^80^ | 2015 | Moderate | Low | Low | Low | Low | Low | Low |
| Crespo-Leiro MG^44^ | 2016 | Moderate | Low | Moderate | Moderate | Moderate | Low | Moderate |
| Akwo EA^81^ | 2017 | Low | Low | Low | Low | Moderate | Low | Low |
| Al-Khateeb M^82^ | 2017 | Moderate | Moderate | Moderate | Low | Moderate | Low | Moderate |
| Dokainish H^83^ | 2017 | Moderate | Low | Moderate | Moderate | Moderate | Low | High |
| Farre N (1)^84^ | 2017 | Low | Low | Low | Low | Low | Low | Low |
| Farre N (2)^85^ | 2017 | Moderate | Low | Low | Low | Low | Low | Low |
| Koudstaal S^86^ | 2017 | Low | Low | Moderate | Low | Moderate | Low | Moderate |
| Mamas MA^87^ | 2017 | Low | Low | Moderate | Low | Moderate | Low | Low |
| Pascual-Figal DA^88^ | 2017 | Moderate | Low | Low | Low | Low | Low | Low |
| Taylor CJ^9^ | 2017 | Low | Low | Moderate | Low | Low | Low | Low |
| Sahle BW^89^ | 2017 | Moderate | Moderate | Moderate | Low | Moderate | Moderate | Moderate |
| Stork S^90^ | 2017 | Low | Low | Moderate | Low | Moderate | Moderate | Moderate |
| Avula HR^91^ | 2018 | Moderate | Low | Low | Low | Moderate | Moderate | Moderate |
| Eriksson B^27^ | 2018 | Moderate | Low | Moderate | Low | Low | Low | Low |
