# Supplementary material for: Survival of patients with chronic heart failure in the community: a systematic review and meta‐analysis
Source: Eur J Heart Fail. 2019 Sep 16;21(11):1306–25. doi: 10.1002/ejhf.1594 (PMC6919428; doi:10.1002/ejhf.1594)
Supplement: Supplementary file 6 — Table S4. GRADE risk of bias assessment across studies. [file EJHF-21-1306-s004.docx]

**Supplementary table 4. GRADE risk of bias assessment across studies**

| **No. of studies** | **Certainty assessment** | | | | | | **Effect** | | | **Certainty** | **Importance** |
| --- | --- | --- | --- | --- | --- | --- | --- | --- | --- | --- | --- |
|  | **Study design** | **Risk of bias** | **Inconsistency** | **Indirectness** | **Imprecision** | **Other considerations** | **No. of events** | **No. of individuals** | **Rate (95% CI)** |  |  |
| **Survival rates at 1-year follow-up (assessed with proportion of patients alive at 1-year)** | | | | | | | | | | | |
| **43** | **Observational studies** | **Not serious** | **Serious ^a^** | **Serious ^b^** | **Not serious** | **Very strong association** | **633931** | **743567** | **Event rate 85.4 per 100 person year(s) (84.1 to 86.5)** | **++++**  **HIGH** | **IMPORTANT** |
| **Survival rates at 5-year follow-up (assessed with proportion of patients alive at 5-years)** | | | | | | | | | | | |
| **31** | **Observational studies** | **Not serious** | **Serious ^a^** | **Serious ^b^** | **Not serious** | **Very strong association** | **238939** | **497768** | **Event rate 52.4 per 100 person year(s)**  **(50.6 to 54.2)** | **++++**  **HIGH** | **IMPORTANT** |

**Explanations**

1. **I^2^ scores are high for survival estimates at each time point, suggesting significant heterogeneity between studies. In part this reflects the narrow confidence intervals of individual studies, which include large sample sizes. However, the I^2^ score also reflects the variation between studies in terms of important variables such as setting, date of recruitment, age of participants and compliance with treatment.**
2. **The pooled analysis includes studies from across the past 70 years and outcomes have improved over this time. Results for participants recruited into prospective studies in the 1950s may not be directly applicable to current populations.**
