# Supplementary material for: Survival of patients with chronic heart failure in the community: a systematic review and meta‐analysis
Source: Eur J Heart Fail. 2019 Sep 16;21(11):1306–25. doi: 10.1002/ejhf.1594 (PMC6919428; doi:10.1002/ejhf.1594)
Supplement: Supplementary file 7 — Table S5. Subgroup and meta‐regression analyses by age at diagnosis, setting, left ventricular ejection fraction, and date. [file EJHF-21-1306-s005.docx]

**Supplementary table 5. Subgroup and meta-regression analyses by age at diagnosis, setting, LVEF and date**

| **Heart failure survival rates** | | | | | | | | |
| --- | --- | --- | --- | --- | --- | --- | --- | --- |
| **1 year** | | | | | **5 years** | | | |
|  | **Subgroup analysis** | | **Meta-regression** | | **Subgroup analysis** | | **Meta-regression** | |
|  | **Number of estimates** | **Pooled estimate (95% CI)** | **Mean difference**  **(95% CI)** | **P value** | **Number of estimates** | **Pooled estimate (95% CI)** | **Mean difference**  **(95% CI)** | **P value** |
| **Overall** | 43 | 86.5 (85.4 to 87.6) |  |  | 30 | 56.7 (54.0 to 59.4) |  |  |
|  |  |  |  |  |  |  |  |  |
| **Age at diagnosis** |  |  | -4.1 (-7.1 to -1.1)^$^ | **0.005^$^** |  |  | -13.4 (-18.8 to -8.0)^$^ | **<0.001^$^** |
| <65 (reference) | 10 | 91.5 (88.2 to 94.3) | - | - | 6 | 78.8 (75.5 to 82.0) | - | - |
| 65-74 | 20 | 88.2 (85.6 to 90.5) | -3.7 (-10.1 to 2.6) | 0.31* | 13 | 58.7 (53.4 to 63.9) | -20.4 (32.4 to -8.4) | 0.002* |
| 75+ | 21 | 83.3 (81.8 to 84.9) | -8.1 (-14.4 to -1.8) | 0.008* | 18 | 49.5 (46.3 to 52.7) | -29.4 (-40.9 to -17.9) | <0.001* |
|  |  |  |  |  |  |  |  |  |
| **Study setting** |  |  |  | **0.06** |  |  |  | **0.047** |
| Primary care (reference) | 17 | 84.9 (81.8 to 87.7) | - | - | 12 | 55.0 (50.8 to 59.2) | - | - |
| Secondary care (Cardiology) | 15 | 89.7 (87.1 to 92.1) | 6.28 (0.2 to 12.3) | 0.02* | 7 | 68.4 (58.9 to 77.2) | 13.4 (-0.4 to 27.2) | 0.06* |
| Secondary care  (Mixed) | 2 | 93.2 (91.8 to 94.6) | 10.0 (-2.6 to 22.7) | 0.99* | n/a | n/a | n/a | n/a |
| Cross-discipline | 10 | 83.3 (81.6 to 85.0) | -1.1 (-7.8 t0 5.7) | 0.30* | 11 | 50.6 (46.3 to 55.0) | -3.9 (-16.1 to 8.2) | 0.85* |
|  |  |  |  |  |  |  |  |  |
| **LVEF** |  |  |  | **0.26** |  |  |  | **0.0007** |
| HFrEF (reference) | 15 | 88.2 (84.1 to 91.8) | - | - | 12 | 63.0 (55.6 to 70.2) | - | - |
| HFmrEF | 2 | 86.4 (85.5 to 87.3) | 0.3 (-12.1 to 12.7) | 0.99* | 2 | 50.1 (48.8 to 51.5) | -8.3 (-25.8 to 9.1) | 0.65* |
| HFpEF | 13 | 89.3 (85.3 to 92.8) | 0.92 (-6.22 to 8.07) | 0.99* | 10 | 69.8 (57.9 to 80.5) | 4.5 (-6.2 to 15.3) | 0.93* |
| Mixed | 24 | 83.5 (82.1 to 84.9) | -4.35 (-10.2 to 1.55) | 0.43* | 17 | 47.0 (44.9 to 49.2) | -15.1 (-24.3 to -5.8) | 0.02* |
|  |  |  |  |  |  |  |  |  |
| **Study date** |  |  | 5.5 (3.2 to 7.8)^$^ | **<0.001^$^** |  |  | 7.8 (2.7 to 12.9)^$^ | **0.013^$^** |
| 1950-1969 | 1 | 70.8 (64.7 to 76.3) | -18.0 (-38.0 to 2.0) | 0.52* | 1 | 35.2 (29.2 to 41.5) | -24.0 (-56.1 to 8.1) | 0.60* |
| 1970-1979 | 2 | 63.1 (59.2 to 66.9) | -25.7 (-40.1 to -11.4) | 0.02* | 2 | 29.1 (25.5 to 32.7) | -29.9 (-52.9 to -6.8) | 0.11* |
| 1980-1989 | 3 | 71.8 (64.5 to 78.6) | -16.2 (-28.3 to -4.1) | 0.13* | 3 | 44.2 (42.3 to 46.2) | -15.9 (-34.5 to 2.8) | 0.43* |
| 1990-1999 | 19 | 84.2 (81.5 to 86.6) | -6.53 (-14.4 to 1.4) | 0.38* | 17 | 56.2 (50.7 to 61.6) | -4.0 (-15.0 to 7.0) | 0.89* |
| 2000-2009 | 21 | 88.5 (86.8 to 90.0) | -1.1 (-8.7 to 6.4) | 0.99* | 12 | 59.7 (54.7 to 64.6) | - |  |
| 2010-2019  (reference) | 4 | 89.3 (84.3 to 93.4) |  |  | n/a | n/a | n/a | n/a |

^$^ Test for trend

^*^comparison with reference category and adjusted for multiple testing using Monte Carlo permutations

n/a: not applicable
