# Supplementary figures and images for: Survival of patients with chronic heart failure in the community: a systematic review and meta‐analysis
Source: Eur J Heart Fail. 2019 Sep 16;21(11):1306–25. doi: 10.1002/ejhf.1594 (PMC6919428; doi:10.1002/ejhf.1594)

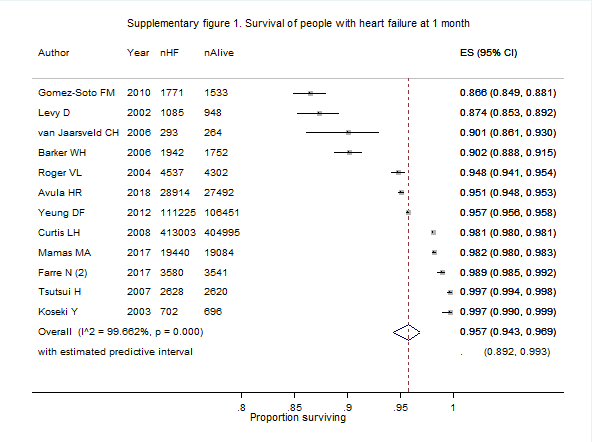

Supplement: Supplementary file 8 — Figure S1. Survival of people with heart failure at 1 month. [file EJHF-21-1306-s006.tif]

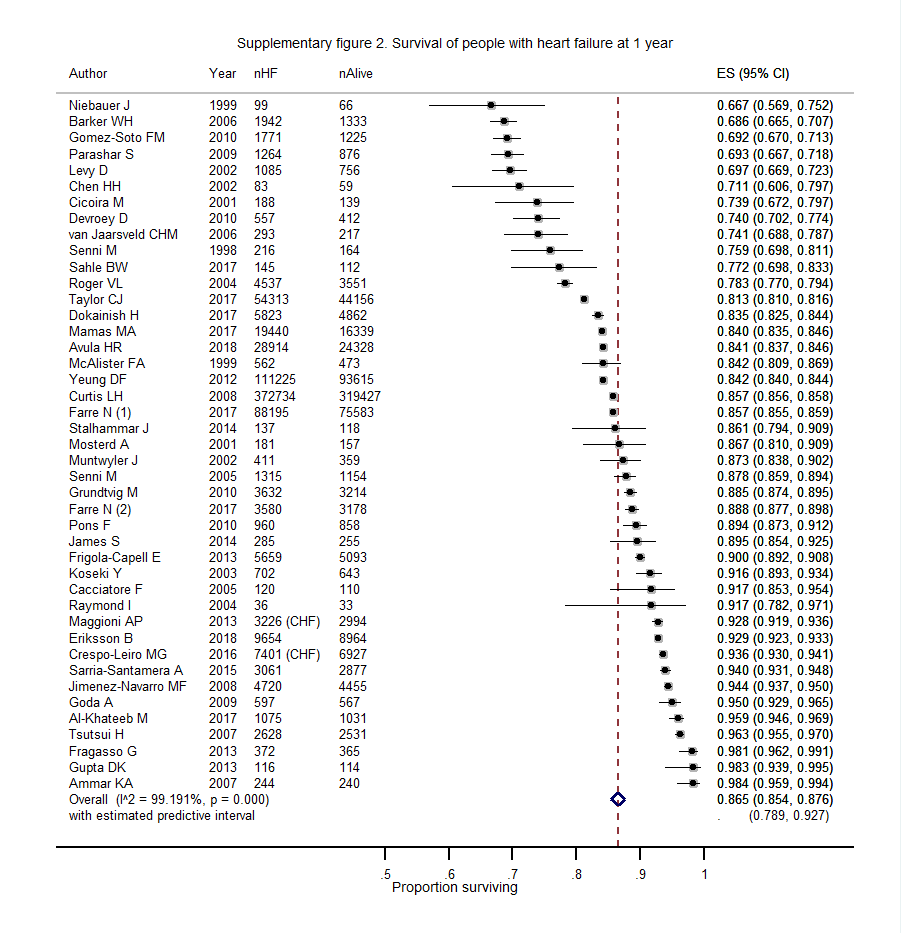

Supplement: Supplementary file 9 — Figure S2. Survival of people with heart failure at 1 year. [file EJHF-21-1306-s007.tif]

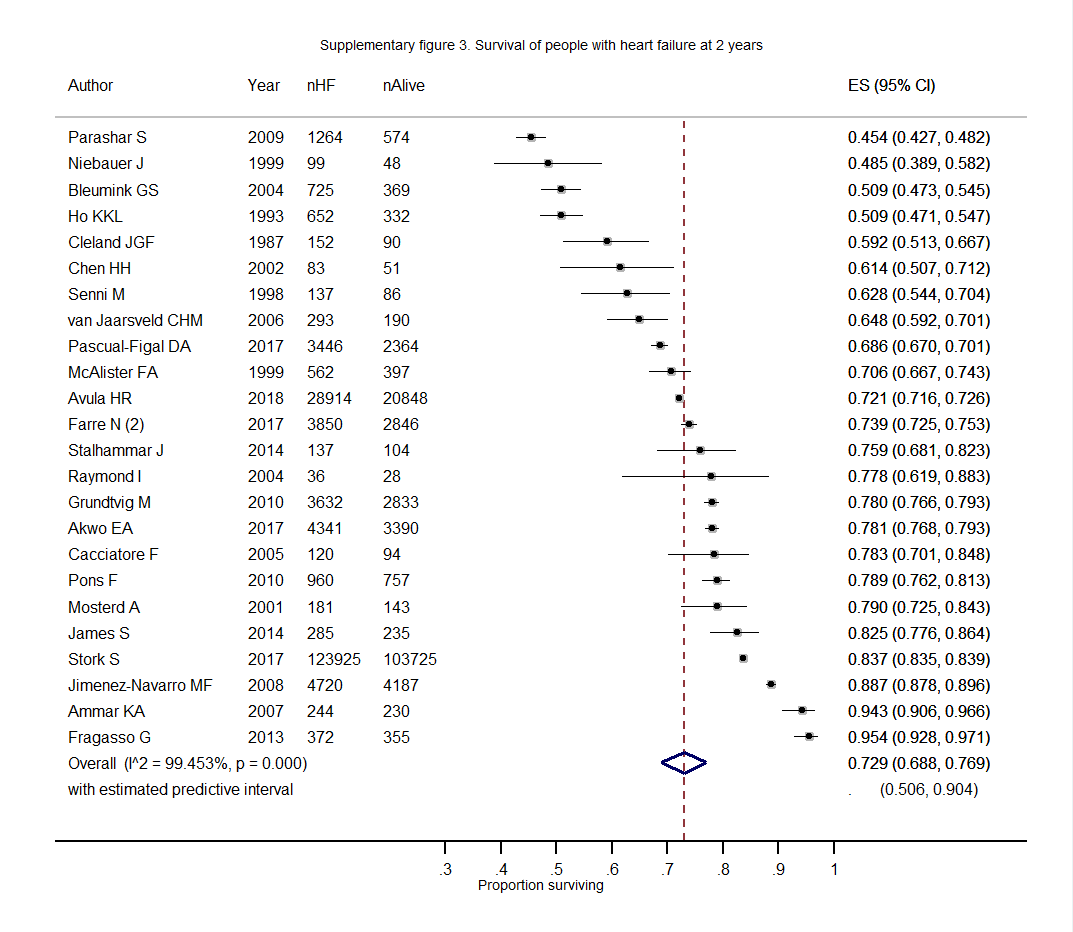

Supplement: Supplementary file 10 — Figure S3. Survival of people with heart failure at 2 years. [file EJHF-21-1306-s008.tif]

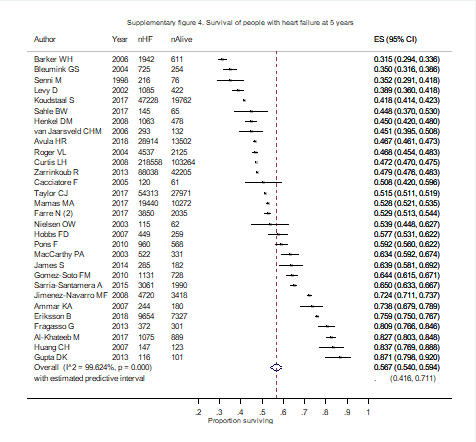

Supplement: Supplementary file 11 — Figure S4. Survival of people with heart failure at 5 years. [file EJHF-21-1306-s009.tif]

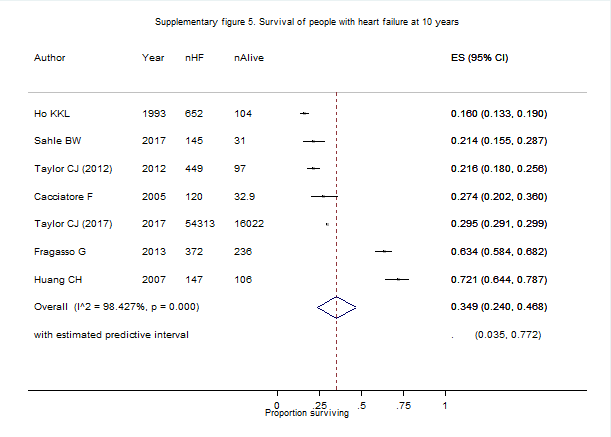

Supplement: Supplementary file 12 — Figure S5. Survival of people with heart failure at 10 years. [file EJHF-21-1306-s010.tif]
