# Supplementary material for: Survival of patients with chronic heart failure in the community: a systematic review and meta‐analysis
Source: Eur J Heart Fail. 2019 Sep 16;21(11):1306–25. doi: 10.1002/ejhf.1594 (PMC6919428; doi:10.1002/ejhf.1594)
Supplement: Supplementary file 13 — Figure S6. PRISMA flow diagram of study selection. [file EJHF-21-1306-s011.docx]

**Supplementary results 2. PRISMA flow diagram of study selection**

Full-text articles excluded, with reasons
(n = 37)

Recruited during hospital admission (n=15)

Short review or conference abstract (n=7)

Overlapping datasets (n=6)

Wrong outcomes (n=6)

Wrong methodology (n=2)

Insufficient data (n=1)

Studies included in quantitative synthesis (meta-analysis)
(n = 58)

Studies included in qualitative synthesis
(n = 60)

Full-text articles assessed for eligibility
(n = 97)

Records excluded
(n = 5326)

Records screened
(n = 5423)

Additional records identified through other sources
(n = 5)

## Identification

## Eligibility

## Included

## Screening

Records identified through database searching
(n = 7118)

Results reported at time points incompatible for meta-analysis (n=2)

Records after duplicates removed
(n = 5423)

Duplicates (n=1700)
